# Editorial: Community series in psychocardiology: exploring the brain-heart interface - Volume II

**DOI:** 10.3389/fpsyt.2024.1438733

**Published:** 2024-06-13

**Authors:** Marlies E. Alvarenga, Don Byrne, Kai G. Kahl

**Affiliations:** ^1^ Institute of Health and Wellbeing, Federation University Australia, Berwick, VIC, Australia; ^2^ Victorian Heart Institute, Melbourne, VIC, Australia; ^3^ School of Medicine and Psychology, Australian National University Canberra, NSW, Australia; ^4^ School of Medicine, Department of Psychiatry. Hannover Medical School, Hanover, Germany

**Keywords:** cardiovascular disease, psychocardiology, psychiatry, brain-heart connection, cardiac psychology

In the intricate web of human health, the connection between mental processes and cardiovascular health is indisputable. A simple Google search of “heart-mind connection research” returns over 400,000 hits, underscoring the breadth and depth of exploration into this nexus. While historical intuition has long hinted at this association, it is only in the past two decades that cardiology, psychiatry, and psychology have begun forging profound and enduring collaborations. Volume I of our discourse (Kahl, Alvarenga & Byrne, 2022, Psychocardiology: Exploring the Brain-Heart Interface, *Frontiers in Psychiatry*, doi. 10 3389/978–2-058–3) laid the groundwork by emphasising the need for further investigation into the brain-heart interface, and thus birthing Volume II of our exploration of this domain.

In Volume 2, Bertele et al. illuminate the significant influence of adverse childhood experiences (ACEs) on adult mental and cardiovascular health, providing evidence that early psychological distress is not confined to the mind but also has the potential to impact onto the body, affecting heart health primarily through lack of exercise and poor dietary choices. They suggest that depression is the mediating factor in the pathway between ACEs and cardiac risk factors, like increased epicardial adipose tissue. This finding carries significant implications for the prevention of heart disease by recognising and addressing mental health issues across the lifespan.

Depression continues to demonstrate its prominence as both a risk factor and a side effect of heart disease, not only amplifying the risk of developing CVD but also worsening its prognosis and complicating its management. Keller-Varady et al.‘s work demonstrates the transformative potential of physical exercise in ameliorating both mental and cardiovascular illness. Their six-week intervention program highlights the increased benefit of adding psychological interventions, such as Motivational Interviewing, to promote a physically active lifestyle, as measured by increased physical fitness following adherence to a physical fitness program. And it would be logical to extrapolate this to an expectation of a positive impact onto cardiovascular health.

In the gerontopsychiatric domain, Schulze-Westhoff et al. investigate the determinants of severe QTc prolongation, which is associated with ventricular dysfunction. The use of antipsychotic drugs in the elderly was linked to QTc prolongation, highlighting the need for tailored approaches in managing psychiatric conditions in elderly individuals. Meanwhile, the management of depression in specific clinical populations, such as cardiac surgery patients, poses unique challenges. Vu and Smith found that depression in cardiac surgery patients seemed to stem from pathophysiological factors such as autonomic nervous system dysregulation, excessive inflammation and disruption of the hypothalamic-pituitary-adrenal axis. Behavioural factors, such as poor diet, insufficient exercise, poor medication compliance and low uptake rates of cardiac rehabilitation also contributed to the development of coronary heart disease in depressed patients. Integrative approaches that combine pharmacotherapy, psychotherapy, and lifestyle interventions hold promise in addressing the complex interplay between depression and cardiovascular health post-surgery.

Posttraumatic stress disorder (PTSD) emerges as another psychological facet intricately linked with heart disease. Using the metacognitive model, Wells et al. examined the prevalence of PTSD in a sample of patients referred to a cardiac rehabilitation program. They found high PTSD in the cardiac rehabilitation sample, and metacognitive beliefs of uncontrollability, worry risk and need to control were linked to both anxiety and depression. The prevalence of post-traumatic stress disorder (PTSD) in patients with coronary heart disease underscores the need for comprehensive screening and intervention strategies, specifically, shedding light on the role of metacognition as a potential intervention for PTSD in this population. Furthermore, by identifying individuals at heightened risk, clinicians can intervene early and mitigate the psychological impact on cardiac illness.

In addition to individual factors, the influence of social determinants, such as relationship status, on the psychological well-being of adults with congenital heart disease cannot be overstated. Social support plays a crucial role, particularly given extensive research linking loneliness to heart disease. Understanding how interpersonal relationships affect mental health is vital for providing comprehensive care to this vulnerable population. Stapel et al. examined the association between relationship status and both anxiety and depression in adults with congenital heart disease, revealing that there are significant impacts. Single individuals exhibited higher depression scores, with single women reporting greater anxiety than single men. This study underscores the advantages of spousal relationships for patients with adult congenital heart disease. Once more, the potential would seem to exist for psychologically based interventions seeking to mitigate the impost of congenital heart disease.

Continuing this theme, Le Grande et al. investigated coping style as a crucial mediator in the relationship between illness knowledge and psychosocial outcomes in women with atrial fibrillation, emphasising the importance of personalised interventions tailored to individual coping mechanisms, and the significant role health care providers need to play in ensuring patients are well versed in the conditions from which they suffer.

Nahlen-Bose undertook a meta-analysis of 67 studies investigating psychosocial interventions in heart failure, revealing the short-term benefit of psychosocial interventions for reducing depression and anxiety and improving quality of life among heart failure patients. Future studies could well focus on the long-term effects of these interventions, not only concerning psychosocial outcomes but also cardiac endpoints.

One of the most promising and innovative approaches to providing psychosocial interventions to cardiac patients involves the use of non-blended web applications as brief metacognitive-based interventions. By leveraging technology, Larionov et al. aimed to deliver accessible and scalable interventions that target cognitive processes underlying emotional distress. Good acceptability and feasibility suggest the potential of these interventions to improving mental well-being and coping strategies in CVD patients, with the expectation of improving cardiac prognosis.

Volume 2 of *Psychocardiology* then, takes up the dominant themes established in Volume 1, providing further evidential weight to an already enticing and persuasive narrative. Those themes, in summary, appear to us to be: first, the prominence of depression in the link between the brain and the heart; second, the importance of considering gender in understanding the brain-heart interface; and third, the enormous potential in translating causal or correlational evidence on the brain-heart interaction into evidence-based interventional strategies addressing both mental and cardiovascular health. And in relation to the last of these themes, the crucial need for intensive and broadly based research into the design, implementation, and evaluation of such intervention strategies. By integrating psychosocial perspectives into cardiovascular care, we can pave the way for improved outcomes and enhanced quality of life for patients facing the dual burden of mental and cardiovascular illness.

## Author contributions

MA: Writing – original draft. DB: Writing – review & editing. KK: Writing – review & editing.

